# California Niño/Niña

**DOI:** 10.1038/srep04801

**Published:** 2014-04-25

**Authors:** Chaoxia Yuan, Toshio Yamagata

**Affiliations:** 1 Application Laboratory, Japan Agency for Marine-Earth Science and Technology, Yokohama 236-0001, Japan

## Abstract

The present study shows the existence of intrinsic coastal air-sea coupled phenomenon in the coastal ocean off Baja California and California in boreal summer for the first time. It contributes significantly to the interannual sea surface temperature (SST) anomalies there. An initial decrease/increase in the equatorward alongshore surface winds weakens/strengthens the coastal upwelling and raises/lowers the coastal SSTs through oceanic mixed-layer processes. The resultant coastal warming/cooling, in turn, heats/cools the overlying atmosphere anomalously, decreases/increases the atmospheric pressure in the lower troposphere, generates an anomalous cross-shore pressure gradient, and thus reinforces or maintains the alongshore surface wind anomalies. The regional air-sea coupled phenomenon seems to be analogous to the well-known El Niño/Southern Oscillation (ENSO) in the tropical Pacific but with much smaller time and space scales, and may be referred to as California Niño/Niña in its intrinsic sense.

Climatological surface winds blow southward all year round along the western coast of Baja California and California and drive the surface water offshore owing to the Ekman transport^1^ originated in the Coriolis force that deflects moving objects to the right of their paths in the Northern Hemisphere. To compensate for the surface water, cold and nutrient-rich subsurface water upwells and increases biological productivity in the coastal surface layer, which supports abundant marine species at different trophic levels. In some years, however, the upwelling is reduced (enhanced) and thus the coastal ocean becomes warmer (colder) than usual^2^,^3^,^4^,^5^. For example, the El Niño years of 1957/58, 1976/77, 1982/83 and 1997/98 witnessed pronounced warming in the northeast Pacific; the anomalous warming lasted for over one season with the maximum monthly SST anomalies up to 2–6°C^2^,^3^,^4^,^5^. The anomalous warming caused general decline in biomass of phytoplankton and zooplankton and intrusion of warm-water species into higher-than-usual latitudes of habitats^6^,^7^,^8^,^9^. Extreme climate was also observed in the nearby continent; increased winter storms hit California in 1982/83 and resulted in one of the wettest winters in record. In contrast, the coastal ocean was extremely cold in the 1999 La Niña year and California experienced cooler-than-normal summer^10^. Researches on the frequent co-occurrences of the tropical ENSO and the coastal warming/cooling have shown that ENSO can influence the coastal ocean via atmospheric teleconnections that modify strength of the alongshore surface winds^4^,^11^,^12^ and/or via coastally trapped Kelvin waves that propagate poleward from the eastern tropical Pacific and influence the coastal thermocline depth, upwelling and SSTs^13^. However, not all of the coastal warming/cooling events are associated with ENSO. The basin-wide atmospheric circulation anomalies in mid-latitudes related to the Pacific Decadal Oscillation and the Aleutian Low are also regarded as contributors to the coastal SST variations^12^,^14^,^15^.

In general, subtropical oceans are passively forced by the overlying atmosphere and have weak influences on the atmosphere, compared to the atmospheric internal variability, in seasonal to interannual time scales^16^. However, the SST anomalies in the coastal upwelling ocean may affect the land-sea thermal contrast and cross-shore pressure gradient in the lower troposphere, thus influencing the strength of the alongshore surface winds. This hypothesis associated with the feedback between the coastal upwelling and alongshore surface winds is adopted to partly explain the seasonal intensification of subtropical highs in summer in the Northern Hemisphere^17^. Recently, the coastal ocean-atmosphere coupled process is named the coastal Bjerknes feedback and applied to explain the regionally intensified SST variability in the coastal ocean off western Australia^18^. Here, we demonstrate that the same coastal Bjerknes feedback contributes significantly to the coastal SST variations off Baja California and California in boreal summer and identify the existence of a regional coupled mode as the California Niño/Niña in relation to the pioneering work of Simpson (1983) among others that describe the warming events in the coastal ocean off western North America as California El Niño^3^,^19^. The important difference from previous works, however, is that we demonstrate the existence of an intrinsic regional coupled mode that is independent of ENSO events.

In the present study, we adopt Monthly National Oceanic and Atmospheric Administration (NOAA) Optimum Interpolation Sea Surface Temperature (OISST) version 2^20^, Pacific Fisheries Environmental Laboratory (PEFL) derived upwelling indices^21^, and National Centers for Environmental Prediction (NCEP)/National Center for Atmospheric Research (NCAR) reanalysis 1^22^ for the period of January 1982 to December 2011. Monthly averaged anomalies in SSTs, upwelling indices and atmospheric variables are calculated by removing the mean seasonal cycles. As shown in Fig. 1a, the coastal SSTs off Baja California show substantial interannual variations. The spatial distribution of monthly standard deviation of SST is similar to the first mode of empirical orthogonal function (EOF) analysis that explains ~47% of total variances (Fig. 1b). The California Niño/Niña index is defined as the SST anomalies averaged over 110°W–120°W and 20°N–30°N (the enclosed coastal ocean in Fig. 1a) where they show the largest interannual variability. In the past three decades, the California Niño/Niña index (Fig. 1c) is significantly correlated with the time series of the first EOF mode with the correlation coefficient up to 0.9, significant at a 99.99% confidence level by the two-tailed *t* test. Hence, both time series represent almost the same interannual SST anomalies off Baja California and California.

Seasonally stratified standard deviations of the California Niño/Niña indices show strong interannual variations from boreal summer to winter. As mentioned above, the California Niño/Niña is strongly influenced by the tropical ENSO. However, the influences are much stronger in boreal winter than summer; the correlation coefficient between January-March (July-September, JAS) Niño3 and California Niño/Niña indices is ~0.65 (~0.32) in the past 30 years, significant at the 99.99% (90%) confidence level by the two tailed *t* test. If the interannual variations of the California Niño/Niña indices simultaneously related to ENSO are linearly regressed out, the California Niño/Niña shows the highest interannual variability in boreal summer. Since the mixed-layer depth is shallower and the isotherms at the surface and in the upper ocean off Baja California are more densely packed in boreal summer than winter (Supplementary Fig. 1), the highest interannual variability in boreal summer may be more closely related to the possible coastal Bjerknes feedback. The standard deviation and first EOF mode based on the JAS SST anomalies (Supplementary Fig. 2) show spatial patterns almost identical to those based on the monthly SST anomalies for the whole calendar year (Fig. 1a–b) except that the first EOF mode of the former explains a little higher percentage of total variances. Hence, hereafter, we focus only on the California Niño/Niña in boreal summer (JAS) and investigate the above hypothesis in this report.

## Results

As shown in Fig. 2a, the anomalous negative sea level pressure (SLP) and cyclonic atmospheric circulation appear off the coast of California three months before the peak phase of the California Niño. The associated alongshore component of the surface wind anomalies is poleward and thus reduces the year-round coastal upwelling and offshore Ekman transport, resulting in the significant positive SST anomalies in the coastal ocean. Over the anomalous warm coastal ocean, air parcels in the planetary boundary layer (PBL) are thus anomalously heated (Fig. 3a). This causes negative SLP anomalies that maintain/enhance the anomalous cross-shore pressure gradient in the lower troposphere and the poleward alongshore surface wind anomalies, which in turn raises the coastal SSTs. The close coupling between the lower troposphere and the coastal ocean during the development phase of California Niño can be clearly seen in Fig. 4a, where the lead-lag correlation coefficients between SST, alongshore surface wind and upwelling indices are plotted. It is shown that at the peak time, the correlation coefficient between the upwelling (also the alongshore wind) and California Niño/Niña indices amounts to −0.7, demonstrating clearly that the coastal Bjerknes feedback contributes to about a half of the total variances of the California Niño/Niña in boreal summer. The California Niño/Niña may be also influenced by the tropical ENSO in boreal summer. Hence, significant SST anomalies can be seen in the eastern tropical Pacific as well in the correlation figures (Fig. 2a). However, we stress that the close regional air-sea coupling off Baja California during the developing phase exists clearly even after the ENSO influences are linearly removed (Figs. 2–4b).

The climatological equatorward surface winds are weakened during the developing phase of the California Niño, but possible contributions from the latent heat flux anomalies to the positive SST anomalies averaged in the region of our interest are limited (Supplementary Figs. 3–4). This is probably because of the spatial inconsistence of the latent heat flux anomalies. In the inshore ocean, the anomalously warm SSTs due to oceanic processes may offset or even surpass the effect of the reduced wind speed on evaporation through changes of the near-surface specific humidity; the latent heat flux anomalies there are insignificant until two months before the peak, and then become significantly increased from the inshore ocean to the overlying atmosphere. In contrast, the latent heat flux anomalies contribute significantly to the positive SST anomalies in the further offshore ocean particularly at the southern edge of the SLP anomalies where the anomalous cyclonic surface winds weaken the climatological anticyclonic surface winds and thus reduce the latent heat fluxes from the ocean to the atmosphere (Fig. 2, Supplementary Figs. 1, 4). In addition, the subtropical ocean is known to be covered by low stratus clouds due to trade wind inversion related to subsidence of the subtropical high^23^. The SST-stratus cloud feedback, through which the warmer-than-normal SST reduces the amount of stratus clouds, increases the downward shortwave radiation and thus enhances the warm SST anomalies, may also contribute to the positive SST anomalies during the developing phase of the California Niño^24^ (Supplementary Fig. 4). However, the surface thermal forcing as a whole may play a secondary role compared to the oceanic processes on the SST anomalies related to California Niño during the developing phase; the highest correlation coefficient between the net surface heat flux anomalies averaged over the region of our interest and the summer California Niño/Niña indices is ~0.4 at most when the California Niño/Niña indices lag two months for the case where the linear ENSO influences are excluded (Supplementary Fig. 3b). In contrast, during the decaying phase, the accumulated positive SST anomalies off Baja California and California are dampened quickly by the surface heat fluxes, in particular the latent heat fluxes, to the overlying atmosphere as shown by much higher correlation coefficients with the California Niño/Niña indices (Supplementary Figs. 3–4).

The anomalous oceanic advection may also contribute to the development of California Niño^3^. The poleward alongshore surface wind anomalies off Baja California during the developing phase of California Niño may reduce the southward transportation of cold water by the California Current, i.e., the equatorward wind-driven surface current^25^. The wind anomalies decrease the offshore Ekman transport and increase the coastal sea surface heights (SSHs) (Supplementary Fig. 5). The anomalous cross-shore gradient of SSHs thus formed is consistent with the poleward surface current anomalies. These processes are comparable to the zonal advective feedback in the tropical Pacific during the ENSO events^26^. Hence, in a broad sense, the coastal ocean is analogous to the tropical ocean as pointed out first by Japanese oceanographer Kozo Yoshida and Chinese oceanographer Han-Lee Mao in collaboration about a half century ago at the Scripps Institution of Ocenography^27^,^28^ when they found a similarity between the equatorial upwelling and the coastal upwelling as the ocean responses to the surface winds. Here we have extended the similarity to ocean-atmosphere coupled processes.

## Discussion

Although the generation mechanism of the California Niño/Niña in boreal summer is quite similar to that of the tropical ENSO, to recognize differences is important, too. The former has much smaller temporal and spatial scales; it is confined to the coastal ocean and lasts only for 1–2 seasons (Figs. 2–3). This is probably because, besides the coastal upwelling, the shallow mixed-layer and sharp vertical and horizontal temperature gradients in the upper ocean are required for the coastal Bjerknes feedback to operate effectively. Therefore, the California Niño/Niña generated by the coastal Bjerknes feedback is seasonally locked to boreal summer within the coastal ocean in the subtropical transition zone. Also, we note that the nature of California Niño/Niña in boreal summer on which we have focused in this study differs from that of California Niño/Niña in boreal winter^2^,^3^,^4^,^5^,^19^. The latter is generally referred to the coastal ocean warming/cooling in the northeast Pacific along almost the entire western coast of North America. The broadly-extended coastal SST anomalies are attributed mainly to the external forcing of the tropical ENSO via both oceanic and atmospheric teleconnections and/or the ENSO-independent basin-scale atmospheric circulation anomalies in mid-latitudes related to the Aleutian Low. Even if the coastal Bjerknes feedback discussed here plays a role, it must be much less. Actually, the correlation coefficient between January-March California Niño/Niña and alongshore wind (upwelling) indices as defined in this study is only about −0.27 (−0.16).

Since the coastal Bjerknes feedback can operate theoretically in the subtropical coastal oceans in summer, we expect that similar coastal Niño/Niña phenomena may exist along the eastern boundaries of the subtropical oceans under subtropical highs in both Northern and Southern Hemispheres. In the austral summer of 2010/11, extreme coastal warming took place off the western coast of Australia and devastated dramatically the marine ecosystem. This anomalous warming was called Ningaloo Niño^29^. Although it was attributed mainly to the oceanic and atmospheric teleconnections related to the co-occurring La Niña in the tropical Pacific, it may have been amplified by the coastal Bjerknes feedback^18^,^29^,^30^.

The California Niño/Niña lasts more than one season and may certainly impact the local marine ecosystem. For example, the spawning and meridional migration of Pacific sardine in the California Current Ecosystem may be influenced by the related SST anomalies^31^, and its abundance may be impacted by the upwelling-induced anomalies in the biological production^32^. Also, the California Niño/Niña may increase/decrease the summer precipitation in Baja California and surface temperature in the nearby coastal continent (Supplementary Fig. 6). Hence, accurate seasonal prediction of the anomalous events could be beneficial to the industrial management and associated economic activities. Since the California Niño/Niña in boreal summer is less influenced by ENSO than that in boreal winter, its seasonal prediction is challenging. So far, the seasonal predictability of subtropical SST anomalies is mostly originated from their robust relationship with the tropical ENSO^33^,^34^,^35^. For instance, the 2010/11 Ningaloo Niño can be successfully predicted nine months ahead in a coupled ocean-atmosphere model by virtue of the co-occurring La Niña^35^. We believe that efforts to predict regional intrinsic climate modes such as California Niño/Niña in boreal summer may contribute to enhancing skills of seasonal prediction for various societal applications as well as to deepening our knowledge of regional climate variations in the extratropics.

## Methods

**Figure 1:**
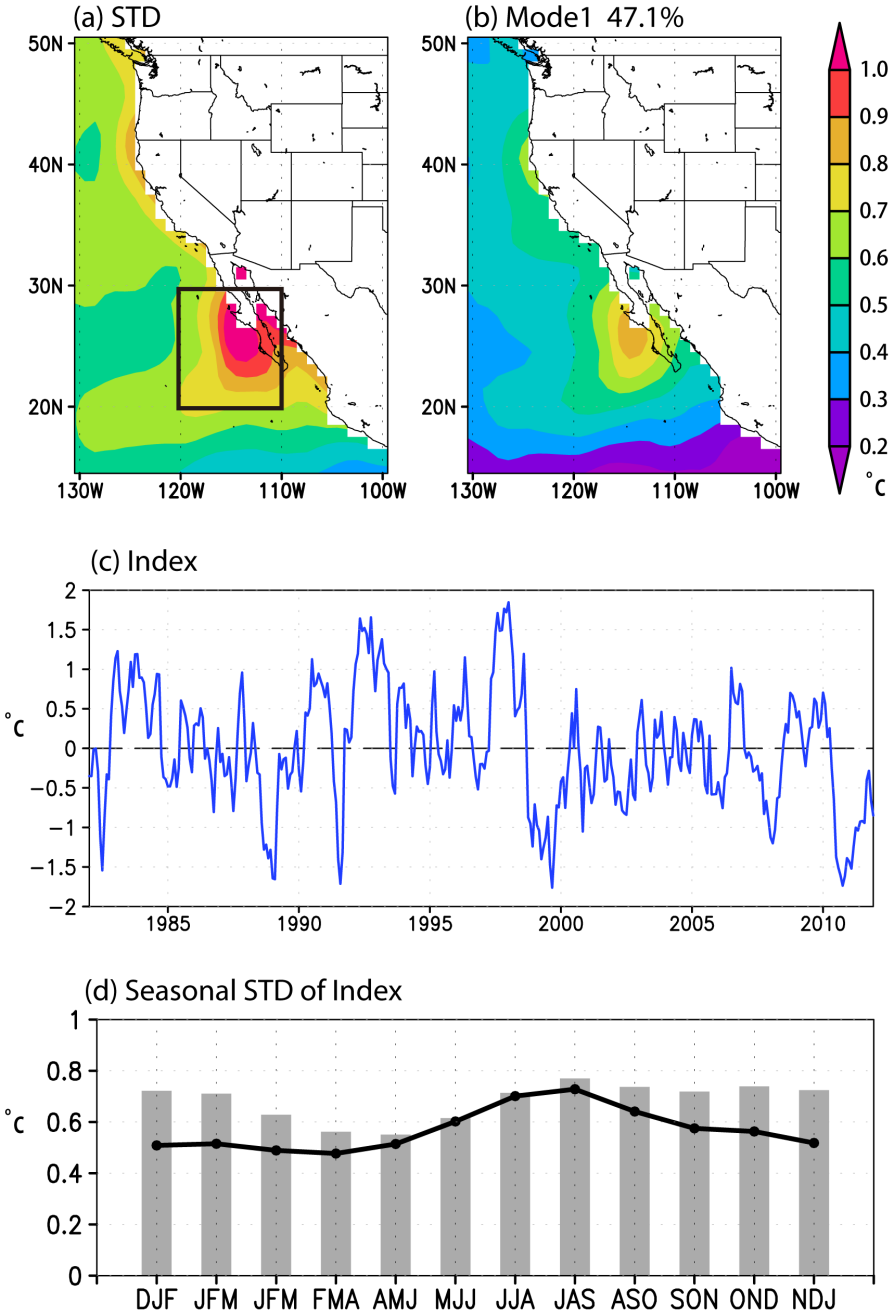
(a) Standard deviation and (b) the first EOF mode of the monthly SST anomalies and (c) the California Niño/Niña indices and (d) their seasonally-stratified standard deviations (grey bar) for the period of January 1982 to December 2011. The enclosed coastal ocean by the dark frame (110°W–120°W, 20°N–30°N) in (a) is the region where the averaged SST anomalies are defined as the indices of California Niño/Niña. The dark line in (d) denotes the seasonally-stratified standard deviations of California Niño/Niña indices after the simultaneous variations related to ENSO are linearly regressed out. The figure was plotted by GrADs software.

We use the National Oceanic and Atmospheric Administration (NOAA) Optimum Interpolation Sea Surface Temperature (OISST) version 2, Pacific Fisheries Environmental Laboratory (PEFL) derived upwelling indices, and National Centers for Environmental Prediction (NCEP)/National Center for Atmospheric Research (NCAR) reanalysis 1 for the period of January 1982 to December 2011. The California Niño/Niña index is defined as the monthly SST anomalies averaged over 110°W–120°W and 20°N–30°N (the enclosed coastal ocean in Fig. 1a). The Niño3 index is the monthly SST anomalies averaged over 90°W–150°W and 5°S–5°N. The JAS California Niño/Niña index with variations related to ENSO linearly regressed out (K) is computed by the formula: 
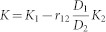
. Here, K_1_ is the JAS California Niño/Niña index, K_2_ the JAS Niño3 index, r_12_ the correlation coefficient between them, D_1_ the standard deviation of K_1_, and D_2_ the standard deviation of K_2_. The alongshore surface wind index is the alongshore component of the 10-meter-height wind anomalies averaged over the same ocean as the California Niño/Niña index after projecting the surface wind along the coast of Baja California (321° to the north). The PEFL upwelling indices are computed from monthly-mean pressure fields and reflect the wind-induced coastal upwelling and off-shore Ekman transport at 15 standard locations along the west coast of North America^21^. Among them, three locations are within the coastal ocean of our interest (24°N 113°W, 27°N 115°W and 30°N 119°W) and thus their monthly anomalies are averaged to represent the upwelling index in this study.

**Figure 2:**
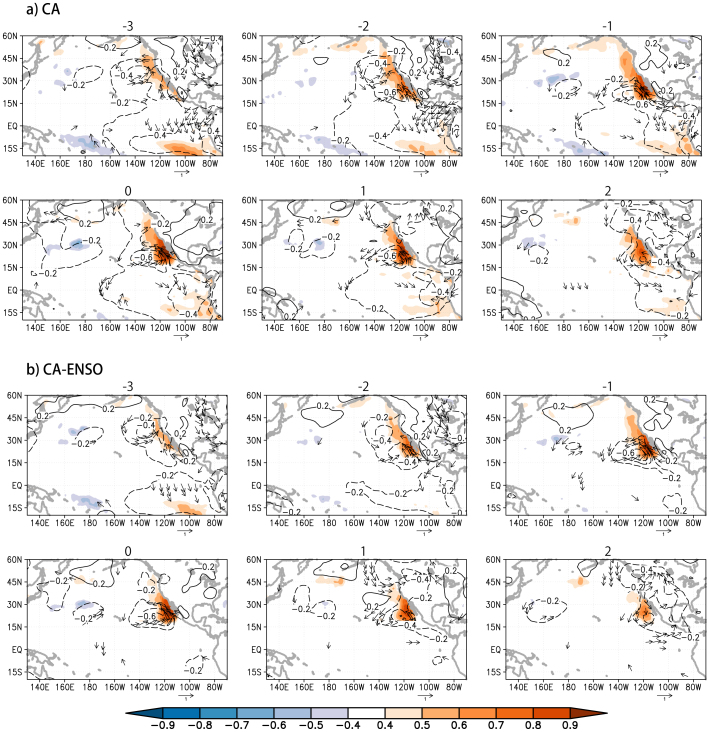
(a) Lead-lag correlation coefficients between the JAS California Niño/Niña indices and 3-month-running mean anomalies in SST (shading), SLP (contour) and 10-meter-height wind (vector). Negative (positive) numbers in the top of each panel denote the months that the JAS California Niño/Niña indices lag (lead). (b) is the same as (a) except that the correlation coefficients are calculated by the JAS California Niño/Niña indices after linearly regressing out the simultaneous variations related to ENSO. Correlation coefficients with SST and wind significant at the 95% confidence level (~0.4) by the two-tailed *t* test are shown only. The figure was plotted by GrADs software.

**Figure 3:**
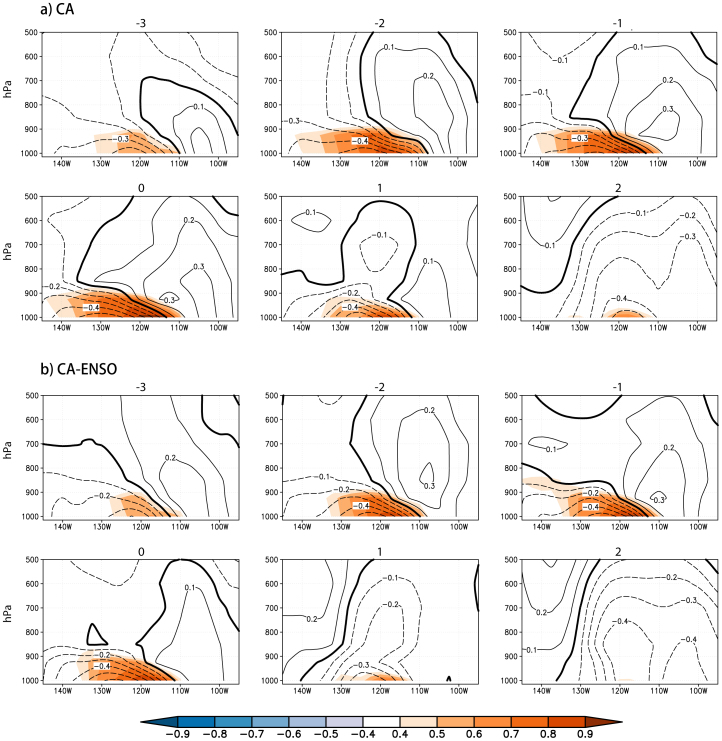
As in Fig. 2, but for the vertical-zonal section of geopotential height (contour) and air temperature (shading) at 25°N. Coefficients with air temperature significant at the 95% confidence level (~0.4) by the two-tailed *t* test are shown only. The figure was plotted by GrADs software.

**Figure 4:**
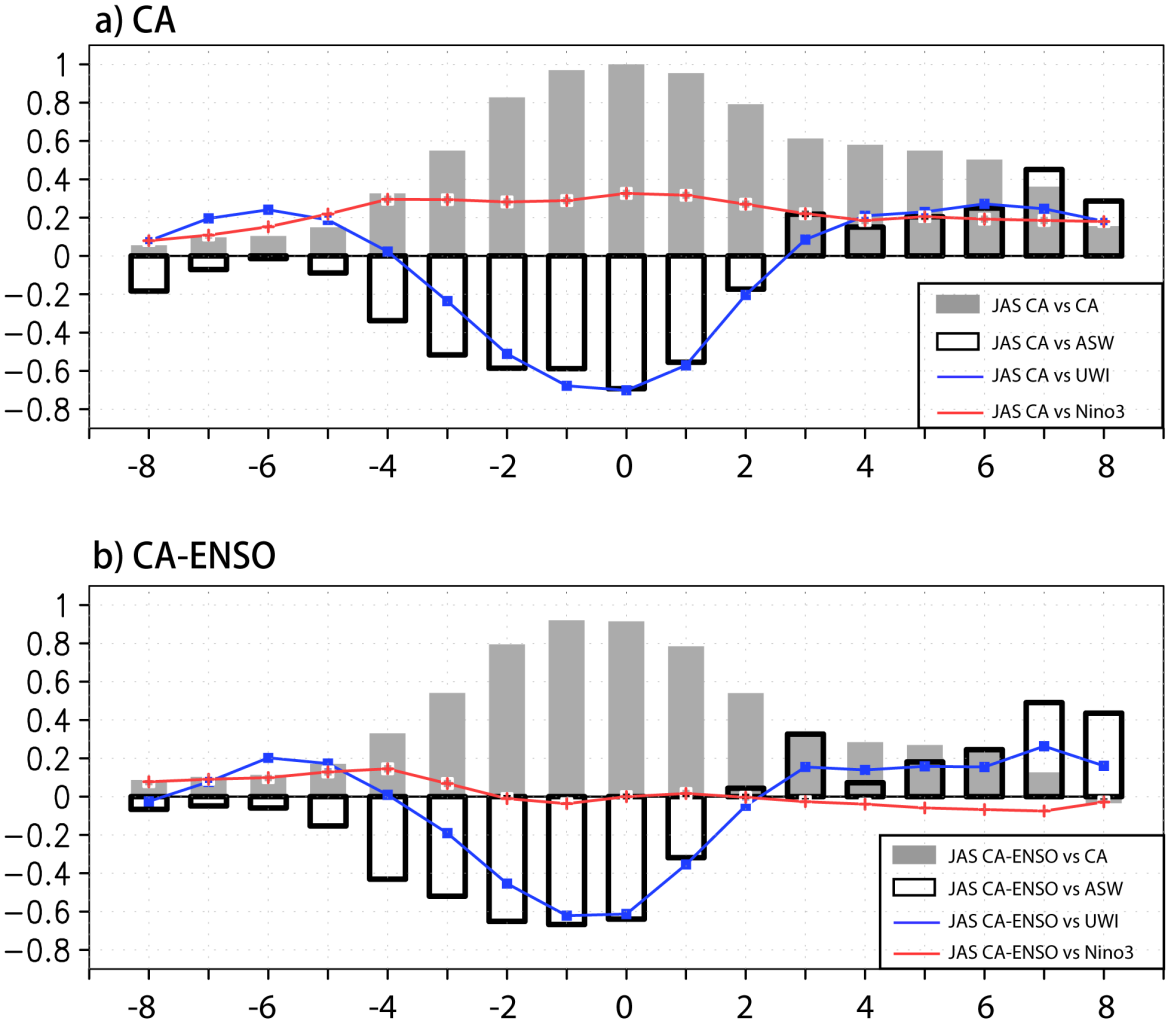
(a) Lead-lag correlation coefficients between the JAS California Niño/Niña indices (JAS CA) and the 3-month-running mean California Niño/Niña (CA, grey filled bar), along-shore surface wind (ASW, dark open bar), upwelling (UMI, blue line) and Niño3 (red line) indices. ASW is positive equatorward. Negative (positive) numbers in the x-axis denote the months that the JAS CA lag (lead). (b) as in (a) except that the correlation coefficients are calculated by the JAS CA after linearly regressing out the simultaneous variations related to ENSO (JAS CA-ENSO). Correlation coefficients of ~0.4 are significant at the 95% confidence level by the two-tailed *t* test. The figure was plotted by GrADs software.

Three-month-running mean anomalies are adopted to represent the monthly anomalies in Figs. 2–4 and Supplementary Figs. 3–6, 8–10 to minimize the intra-seasonal variations, but similar results can be obtained if monthly data are used (Supplementary Fig. 7). The linear correlation and regression analyses are applied in this study to demonstrate the existence of coastal Bjerknes feedback for simplicity. However, there may be asymmetry between California Niño and Niña. For instance, California Niña may have larger anomalous amplitudes and spatial extension than Niño and last for a longer time (Supplementary Figs. 8–10). Also, the JAS California Niño/Niña indices show a negative skewness of −0.11/−0.20 with/without the simultaneous variations linearly related to ENSO. The asymmetry may be partly due to the co-occurrence of negative phase Pacific Decadal Oscillation with California Niña (Supplementary Fig. 8b) and partly due to non-linear processes; this needs to be studied further. We note that the results shown in this study do not change quantitatively even if the NOAA Extended Reconstructed Sea Surface Temperature (ERSST) version 3b, NCEP-National Energy Research Supercomputing Center of the Department of Energy (DOE) reanalysis 2 and European Centre for Medium-Range Weather Forecast Interim Reanalysis (ERA-Int) are used.

## Author Contributions

T.Y. initiated the present research project and C.Y. and T.Y. co-designed the study. C.Y. performed the analyses and prepared the first draft. T.Y. supervised the whole processes.

## Supplementary Material

Supplementary Information supplementary information

## References

[b1] Ekman V. W. On the influence of the earth's rotation on ocean currents. Ark. Mat. Astron. Fys. 2, 1–52 (1905).

[b2] Reid J. L. Oceanography of the eastern North Pacific in the last 10 years. Symposium on The Changing Pacific Ocean in 1957 and 1958, Reports Vol. VII [77–90] (California Cooperative Oceanic Fisheries Investigations, 1960).

[b3] Simpson J. J. Large-scale thermal anomalies in the California Current during the 1982-1983 El Niño. Geophys. Res. Lett. 10, 937–940 (1983).

[b4] Schwing F. B., Murphree T., deWitt L. & Green P. M. The evolution of oceanic and atmospheric anomalies in the northeast Pacific during the El Niño and La Niña events of 1995–2001. Prog. Oceanogr. 54, 459–491 (2002).

[b5] Durazo R. & Baumgartner T. R. Evolution of oceanographic conditions off Baja California: 1997–1999. Prog. Oceanogr. 54, 7–31 (2002).

[b6] Balech E. The changes in the phytoplankton population off the California coast. Symposium on The Changing Pacific Ocean in 1957 and 1958, Reports Vol. VII [127–135] (California Cooperative Oceanic Fisheries Investigations, 1960).

[b7] Roemmich D. & McGowan J. Climatic warming and decline of zooplankton in the California Current. Science 267, 1324–1326 (1995). 17812604 10.1126/science.267.5202.1324

[b8] Chavez F. P. *et al.* Biological and chemical consequences of the 1997–1998 El Niño in central California waters. Prog. Oceanogr. 54, 205–232 (2002).

[b9] Pearcy W. G. Effects of the 1997–98 El Niño on marine nekton off Oregon. Prog. Oceanogr. 54, 399–403 (2002).

[b10] Schwing F. B. & Moore C. S. A year without summer for California, or a harbinger of a climate shift? Eos, Trans. Amer. Geophys. Union 81, 301–305 (2000).

[b11] Bjerknes J. Atmospheric teleconnections from the equatorial Pacific. Mon. Wea. Rev. 97, 163–172 (1969).

[b12] Emery W. & Hamilton D. Atmospheric forcing of interannual variability in the northeast Pacific Ocean: Connections with El Niño. J. Geophys. Res. 90, 857–868 (1985).

[b13] Chelton D. B. & Davis R. E. Monthly mean sea-level variability along the west coast of North America. J. Phys. Oceanogr. 12, 757–783 (1982).

[b14] Mysak L. A. El Niño, interannual variability and fisheries in the northeast Pacific ocean. Can. J. Fish. Aquat. Sci. 43, 464–497 (1986).

[b15] Mantua N. J., Hare S. R., Zhang Y., Wallace J. M. & Francis R. C. A Pacific interdecadal climate oscillation with impacts on salmon production. Bull. Amer. Meteor. Soc. 78, 1069–1079 (1997).

[b16] Kushnir Y. *et al.* Atmospheric GCM response to extratropical SST anomalies: synthesis and evaluation. J. Clim. 15, 2233–2256 (2002).

[b17] Miyasaka T. & Nakamura H. Structure and formation mechanisms of the Northern Hemisphere summertime subtropical highs. J. Clim. 18, 5046–5065 (2005).

[b18] Kataoka T., Tozuka T., Behera S. & Yamagata T. On the Ningaloo Niño/Niña. Clim. Dyn. 10.1007/s00382-013-1961-z (2013).

[b19] Norton J., McLain D., Brainard R. & Husby D. The 1982–83 El Niño event off Baja and Alta California and its ocean climate context. El Niño North [Wooster W. S., & Fluharty D. F. (ed.)] [44–72] (Washington Sea Grant Program, University of Washington, Seattle, 1985).

[b20] Reynolds R. W. *et al.* An improved in situ and satellite SST analysis for climate. J. Clim. 15, 1609–1625 (2002).

[b21] Bakun A. Coastal upwelling indices, west coast of North America, 1946–71. U.S. Dept. of Commerce, NOAA Tech. Rep., NMFS SSRF-671 (1973).

[b22] Kalnay E. *et al.* The NCEP/NCAR 40-year reanalysis project. Bull. Amer. Meteor. Soc. 77, 437–471 (1996).

[b23] Klein S. A. & Hartmann D. L. The seasonal cycle of low stratiform clouds. J. Clim. 6, 1587–1606 (1993).

[b24] Clement A. C., Burgman R. & Norris J. R. Observational and model evidence for positive low-level cloud feedback. Science 325, 460–464 (2009). 19628865 10.1126/science.1171255

[b25] Lynn R. J. & Simpson J. J. The California Current system: The seasonal variability of its physical characteristics. J. Geophys. Res. 92, 12947–12966 (1987).

[b26] Jin F. F., Kim S. T. & Bejarano L. A coupled stability index for ENSO. Geophys. Res. Lett. 33, 10.1029/2006GL027221.

[b27] Yoshida K. & Mao H. L. A theory of upwelling of large-horizontal extent. J. Mar. Res. 16, 40–54 (1957).

[b28] Yoshida K. A theory of the Cromwell Current (the equatorial undercurrent) and of the equatorial upwelling-An interpretation in a similarity to a coastal circulation. J. Oceanogr. Soc. Japan 15, 159–170 (1959).

[b29] Feng M., McPhaden J. J., Xie S. P. & Hafner J. La Niña forces unprecedented Leeuwin Current warming in 2011. Sci. Rep. 3, 1277, 10.1038/srep01277 (2013). 23429502 10.1038/srep01277 PMC3572450

[b30] Tozuka T., Kataoka T. & Yamagata T. Locally and remotely forced atmospheric circulation anomalies of Ningaloo Niño/Niña. Clim. Dyn. 10.1007/s00382-013-2044-x.

[b31] Demer D. A. *et al.* Prediction and confirmation of seasonal migration of Pacific sardine (Sardinops sagax) in the California Current Ecosystem. Fishery Bull. 110, 52–70 (2012).

[b32] Jacobson L. D. & MacCall A. D. Stock-recruitment models for Pacific sardine (Sardinops sagax). Can. J. Fish. Aquat. Sci. 52, 566–577 (1995).

[b33] Yuan C., Tozuka T., Luo J. & Toshio Y. Predictability of the subtropical dipole modes in a coupled ocean-atmosphere model. Clim. Dyn. 42, 1291–1308 (2014).

[b34] Wen C., Xue Y. & Kumar A. Seasonal prediction of North Pacific SSTs and PDO in the NCEP CFS hindcasts. J. Clim. 25, 5689–5710 (2012).

[b35] Doi T., Behera S. & Yamagata T. Predictability of the Ningaloo Niño/Niña. Sci. Rep. 3, 2892 10.1038/srep02892 (2013). 24100593 10.1038/srep02892 PMC3792415

