# Supplementary material for: California Niño/Niña
Source: Sci Rep. 2014 Apr 25;4:4801. doi: 10.1038/srep04801 (PMC3999445; doi:10.1038/srep04801)
Supplement: Supplementary Information [file srep04801-s1.pdf]

Supplementary Information

Chaoxia Yuan* and Toshio Yamagata
Application Laboratory, Japan Agency for Marine-Earth Science and Technology,
Yokohama 236-0001, Japan

The data used for the supplementary figures include NCEP/NCAR reanalysis 1, ERA-Int[36], OISST, Global Precipitation Climatology Project monthly precipitation (GPCP)[37] and NCEP Global Ocean Data Assimilation System (GODAS)[38] from January 1982 to December 2011. Objectively Analyzed air-sea heat Fluxes for the Global Oceans (OAFlux)[39] and International Satellite Cloud Climatology Project (ISCCP)[40] from July 1983 to December 2009 are also used. Three-month-running mean anomalies are adopted to represent the monthly anomalies during the linear correlation, regression and composite analyses to minimize the intra-seasonal variations in Supplementary Figs. 3-6, 8-10.

36. Dee, D. P. et al. The ERA-interim reanalysis: configuration and performance of the data assimilation system. *Q. J. R. Meteorol Soc*, **137**, 553-597 (2011)

37. Adler, R. F. et al. The version 2 global precipitation climatology projection (GPCP) monthly precipitation analysis (1979-Present). *J. Hydrometeor.*, **4**, 1147-1167 (2003)

38. Behringer, D. & Xue, Y. Evaluation of the global ocean data assimilation system at NCEP: The Pacific Ocean. Preprints, Eighth Symp. On Integrated Observing and Assimilation System for Atmosphere, Ocean, and Land Surface, Seattle, WA, *Amer. Meteor.*

Soc. (2004)

39. Yu, L. & Weller, R. A. Objectively Analyzed air-sea heat Fluxes (OAFlux) for the global oceans. *Bull. Ameri. Meteor. Soc.* **88**, 527-539 (2007)

40. Rossow, W. B. & Schiffer R. A. Advances in understanding clouds from ISCCP. *Bull. Amer. Met. Soc.* **80**, 2261-2287 (1999)

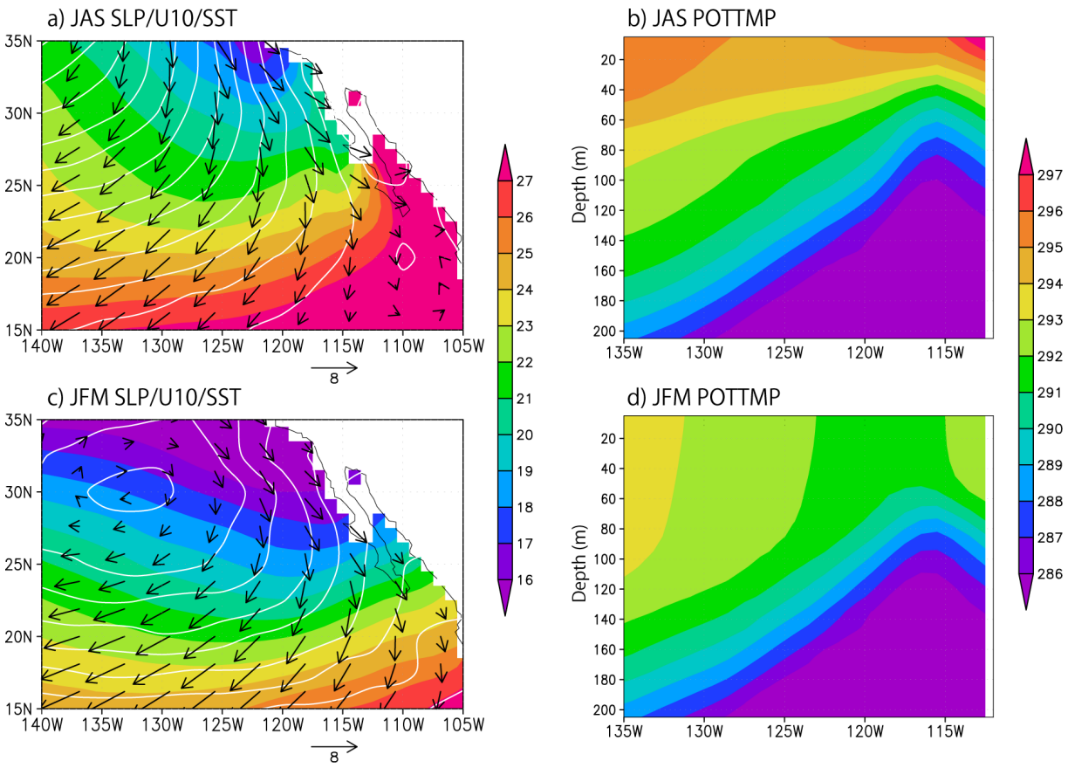

Supplementary Figure 1: Long-term mean (a, c) SLP (contour, hPa), 10-meter-height wind (vector, m s^{-1}) and SST (shading, ˚C) and (b, d) vertical-zonal sections of potential temperature (shading, K) in the upper ocean at 25˚N in (a, b) July-September (JAS) and (c, d) January-March. The figure was plotted by Grads software.

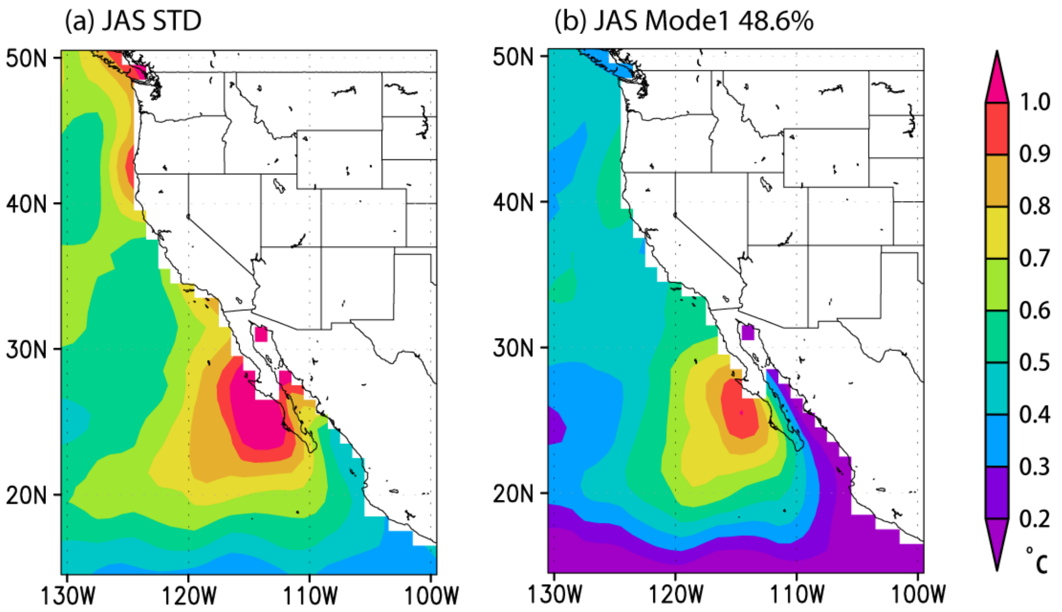

Supplementary Figure 2: (a) Standard deviation and (b) the first EOF mode of the JAS SST anomalies from 1982 to 2011. The explained variance by the first EOF mode is 48.56%. The figure was plotted by GrADs software.

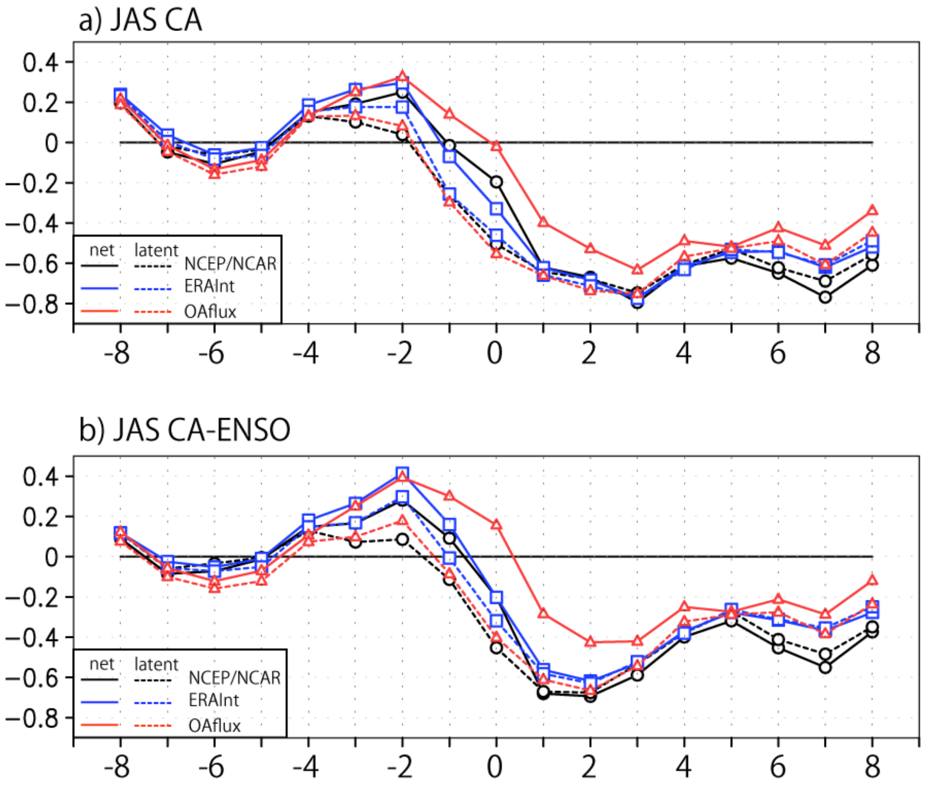

Supplementary Figure 3: (a) Lead-lag correlation coefficients between JAS California Niño/Niña indices and the 3-month-running mean net surface (solid line) and latent (dashed line) heat fluxes averaged over the enclosed coastal ocean in Fig. 1a based on NCEP/NCAR (dark line), ERA-Int (blue line) and OAflux (red line). Negative (positive) numbers in the x-axis denote the months that JAS California Niño/Niña indices lag (lead). (b) as in (a) except that the correlation coefficients are calculated by the JAS California Niño/Niña indices after linearly regressing out the simultaneous variations related to ENSO. Correlation coefficients of ~0.4 are significant at the 95% confidence level by the two-tailed *t* test. The figure was plotted by GrADs software.

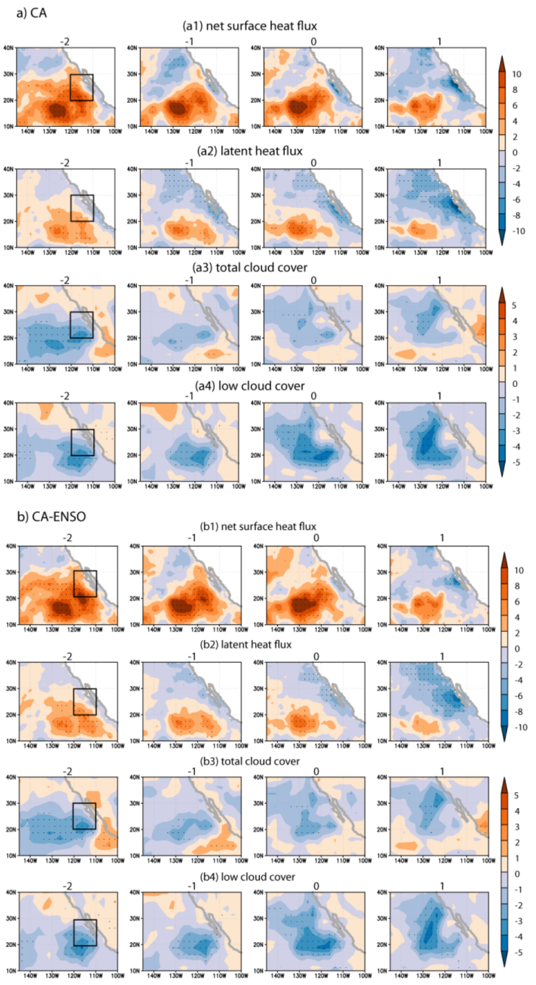

Supplementary Figure 4: (a) Lead-lag linear regression of 3-month-running mean anomalies in (a1) net surface and (a2) latent heat fluxes (W m^{-2}, positive downward), and (a3) total and (a4) low cloud cover percentage based on the JAS California Niño/Niña indices. Negative (positive) numbers on the top of each panel denote the months that the JAS California Niño/Niña indices lag (lead). The dark frames in the first column denote the coastal ocean of interest. (b) as in (a) except that the regression is computed based on the JAS California Niño/Niña indices after linearly regressing out the simultaneous variations related to ENSO. Anomalies significant at the 95% confidence level by the two-tailed *t* test are stippled. OAFlux and ISCCP data are used here. The figure was plotted by GrADs software.

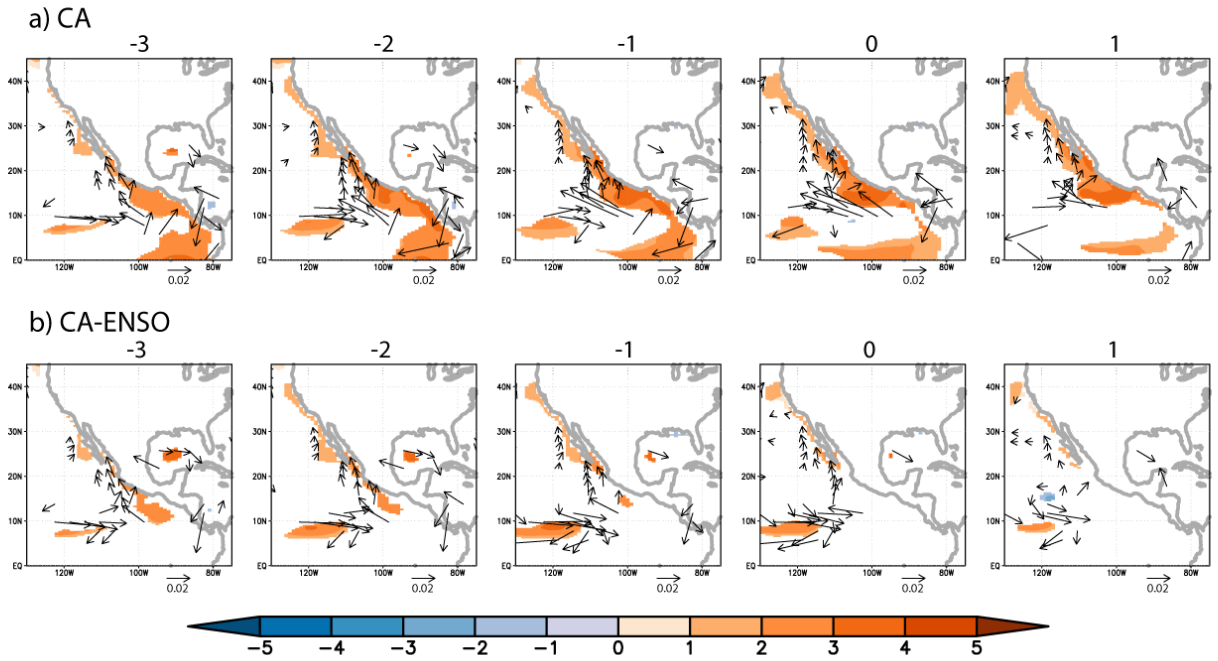

Supplementary Figure 5: Lead-lag linear regression of 3-month-running mean anomalies in sea surface height (shading, mm) and horizontal current at 5 meter depth (vector, m s^{-1}) based on the JAS California Niño/Niña indices. Negative (positive) numbers on the top of panels denote the months that JAS California Niño/Niña indices lag (lead). (b) as in (a) except that the regression is computed based on the JAS California Niño/Niña indices after linearly regressing out the simultaneous variations related to ENSO. Anomalies significant at the 95% confidence level by the two-tailed *t* test are shown only. The figure was plotted by GrADs software.

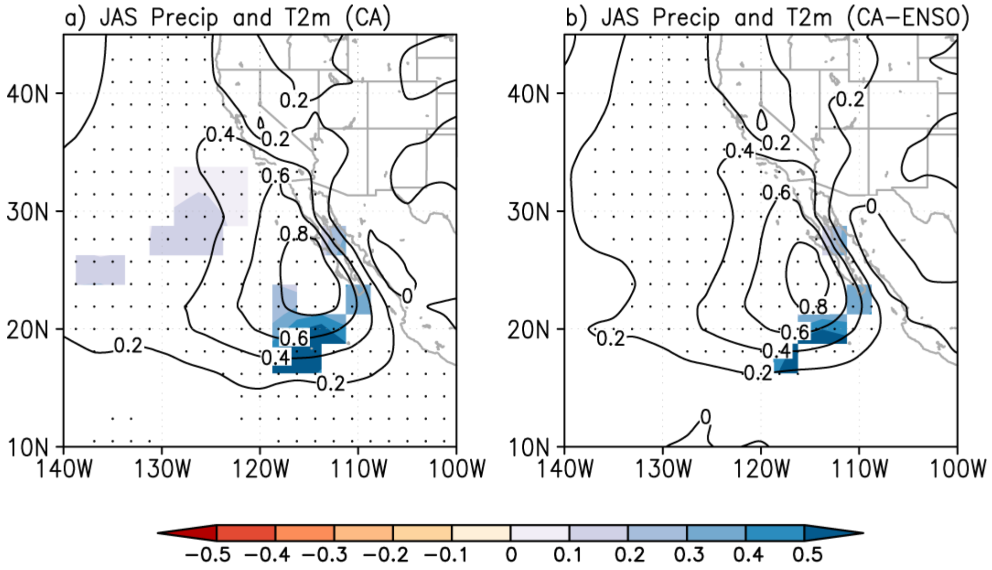

Supplementary Figure 6: (a) Linear regression of anomalies in the JAS precipitation (shading, mm day^{-1}) and 2-meter-high temperature (contour, ˚C) based on the JAS California Niño/Niña indices. (b) as in (a) except that the regression is computed based on the JAS California Niño/Niña indices after linearly regressing out the simultaneous variations related to ENSO. Precipitation (temperature) anomalies significant at the 95% confidence level by the two-tailed *t* test are shown only (stippled). The figure was plotted by GrADs software.

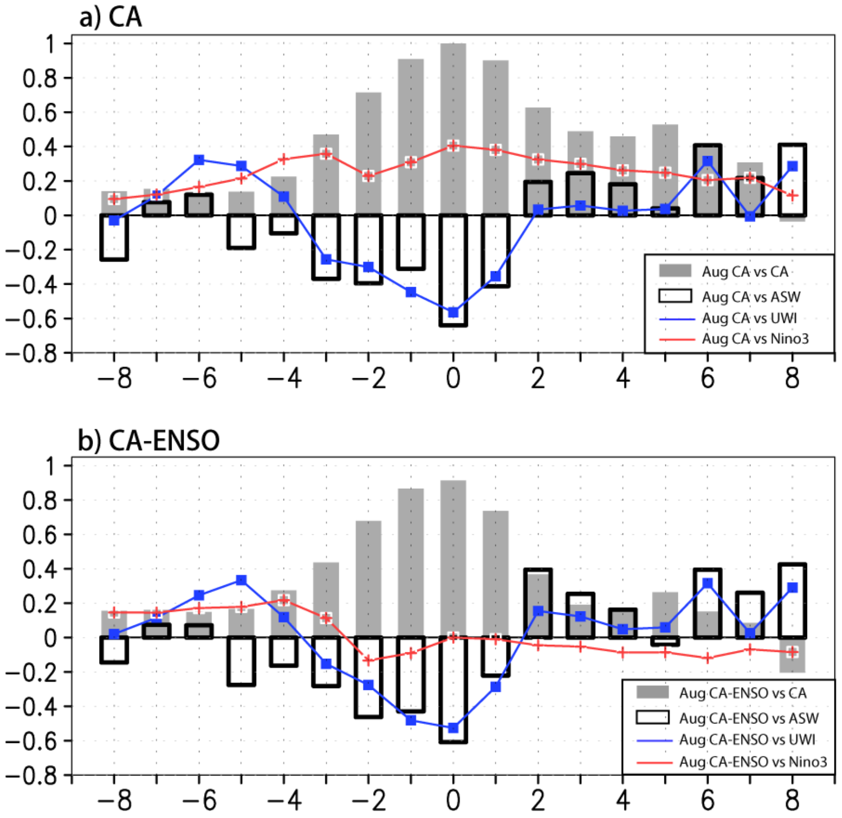

Supplementary Figure 7: a) Lead-lag correlation coefficients between the August California Niño/Niña indices (Aug CA) and the monthly California Niño/Niña (CA, grey filled bar), along-shore surface wind (ASW, dark open bar), upwelling (UMI, blue line) and Niño3 (red line) indices. ASW is positive equatorward. Negative (positive) numbers in the x-axis denote the months that the Aug CA lag (lead). (b) as in (a) except that the correlation coefficients are calculated by the Aug CA after linearly regressing out the simultaneous variations related to ENSO (Aug CA-ENSO). Correlation coefficients of ~0.4 are significant at the 95% confidence level by the two-tailed *t* test. The figure was plotted by GrADs software.

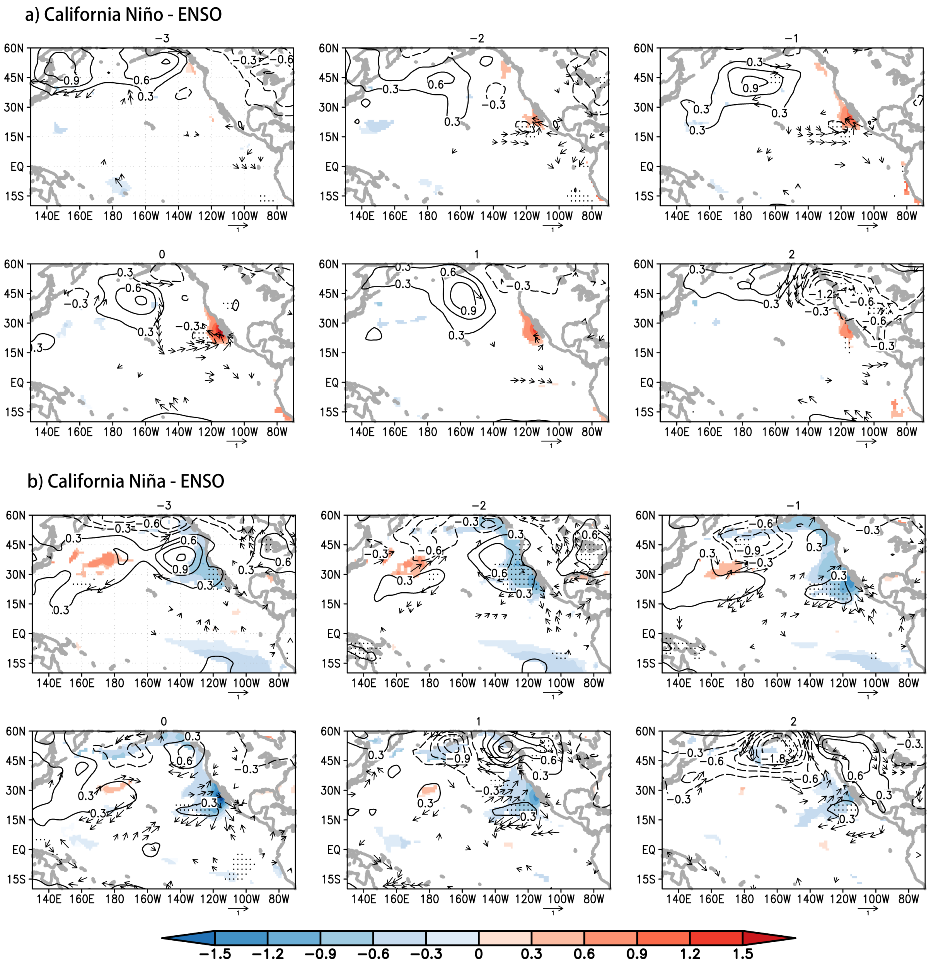

Supplementary Figure 8: Composites of 3-month-running mean anomalies in SST (shading, ˚C), SLP (contour, hPa) and 10-meter-height wind (vector, 1 m s^{-1}) for (a) California Niño and (b) California Niña based on the residual anomalies after linearly removing the ENSO-related simultaneous variations in each anomalous field. Negative (positive) numbers in the top of each panel denote the months that the JAS California Niño/Niña lag (lead). The anomalies in SST and wind (SLP) significant at the 95% confidence level by the two-tailed *t* test are shown only (stippled). The event years are selected if the JAS California Niño/Niña indices after linearly removing the ENSO-related simultaneous variations are above/below 0.5˚C (-0.5˚C). There are eight (seven) California Niño (Niña) years, including 1983, 1984, 1985, 1990, 1992, 1997, 1998 and 2006 (1982, 1987, 1991, 1999, 2002, 2010 and 2011). The figure was plotted by GrADs software.

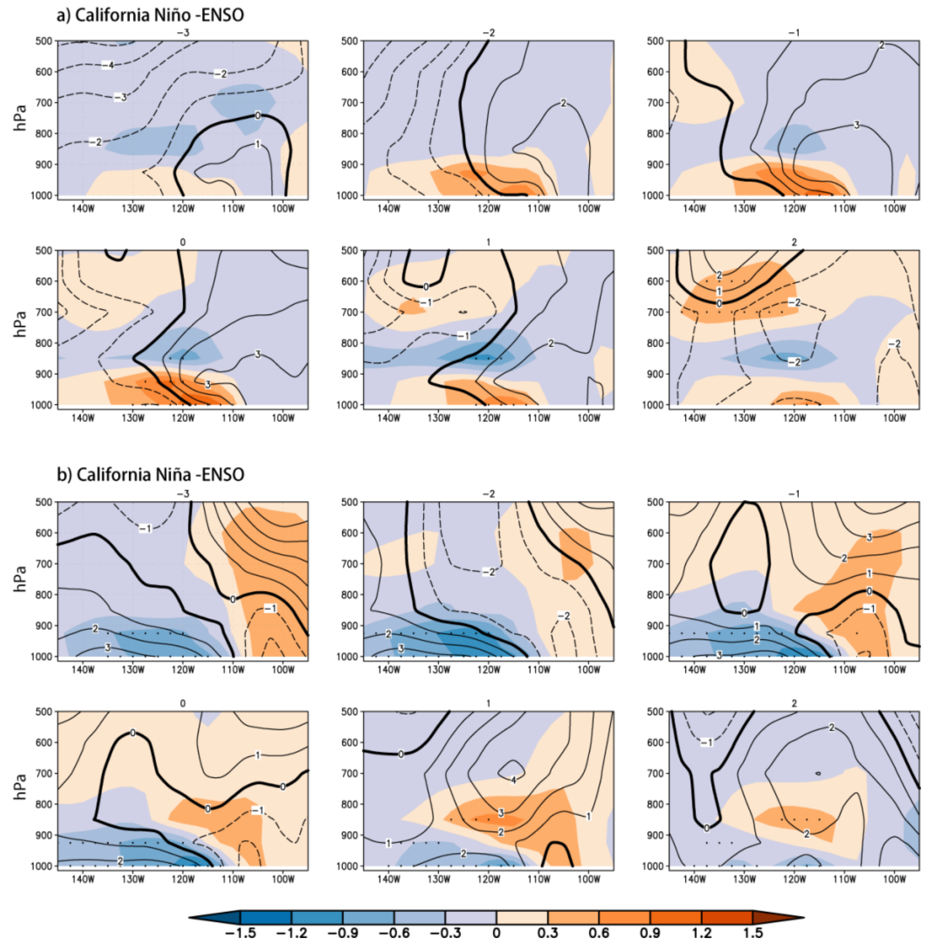

Supplementary Figure 9: As in Supplementary Fig. 8, but for the vertical-zonal section of geopotential height (contour, hPa) and air temperature (shading, ˚C) at 25˚N. Air temperature anomalies significant at the 95% confidence level by the two-tailed *t* test are stippled. The figure was plotted by GrADs software.

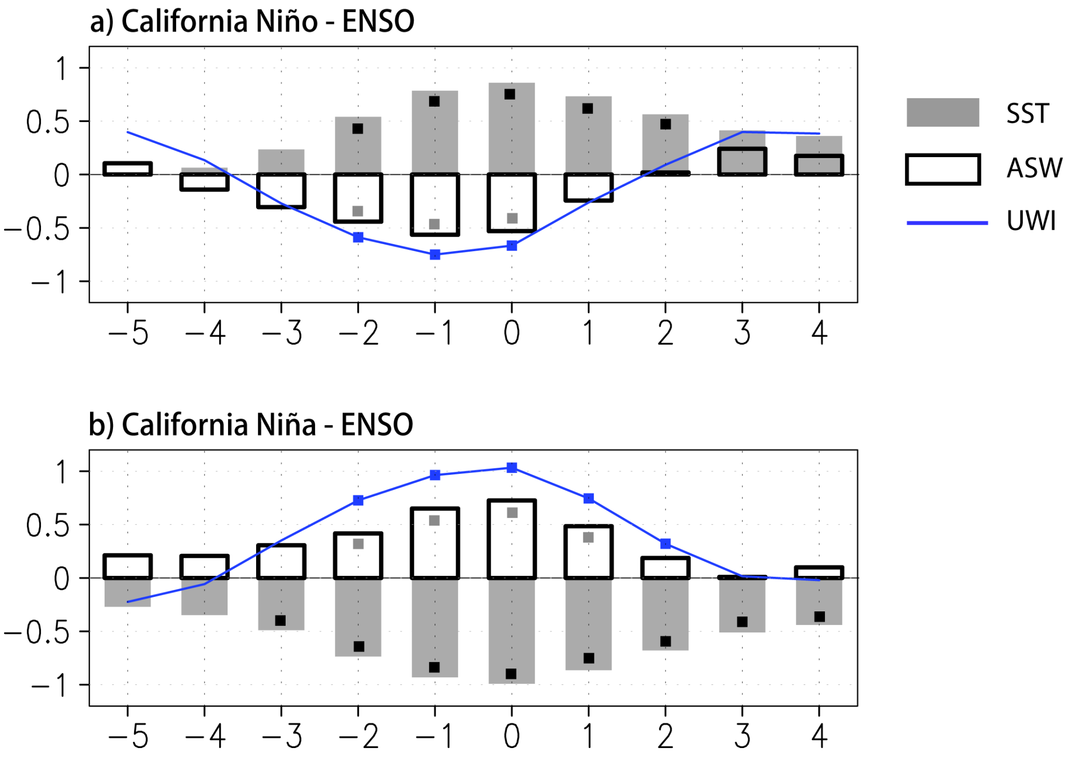

Supplementary Figure 10: As in Supplementary Fig. 8, but for California Niño/Niña indices (grey filled bar, ˚C), ASW (dark open bar, m s^{-1}) and UWI (blue line, m^3 s^{-1} per 100 meters of coastline). The ASW (UWI) has been multiplied by 2 (divided by 20). Anomalies significant at the 95% confidence level by the two-tailed *t* test are marked by the filled squares. The figure was plotted by GrADs software.
